# Gustave Roussy immune score is a prognostic marker in patients with small cell lung cancer undergoing immunotherapy: a real-world retrospective study

**DOI:** 10.3389/fonc.2023.1195499

**Published:** 2023-05-02

**Authors:** Jian Shangguan, Xinyi Huang, Xu Liu, Zengfu Zhang, Xiaodong Zhang, Jinming Yu, Dawei Chen

**Affiliations:** ^1^ Department of Radiation Oncology, Shandong Cancer Hospital and Institute, Shandong First Medical University and Shandong Academy of Medical Sciences, Jinan, Shandong, China; ^2^ Shandong First Medical University and Shandong Academy of Medical Sciences, Jinan, Shandong, China; ^3^ Department of Radiation Oncology, Shandong University Cancer Center, Jinan, Shandong, China

**Keywords:** Gustave Roussy immune score, predictive value, small cell lung cancer, propensity score matching, survival analysis

## Abstract

**Background:**

The utilization of the Gustave Roussy Immune Score (GRIm-Score) in patient selection for immunotherapy was initially reported. The objective of this retrospective study is to assess the potential of the GRIm-Score, a novel prognostic score based on nutritional and inflammatory markers, as a prognostic predictor in patients with small cell lung cancer (SCLC) undergoing immunotherapy.

**Methods:**

This retrospective study conducted at a single center included 159 patients with SCLC who received immunotherapy. The objective of the study was to investigate potential differences in overall survival (OS) and progression-free survival (PFS) among patients stratified by their GRIm-Score, utilizing the Kaplan–Meier survival analysis and the log-rank test. The final independent prognostic factors were identified through both propensity score matching (PSM) analysis and multivariable Cox proportional hazards regression analysis.

**Results:**

Our analysis of the 159 patients revealed that there was a significant decrease in both OS and PFS with each increase in the GRIm-Score group, displaying a stepwise pattern. Moreover, even after conducting PSM analysis, the significant associations between the modified three-category risk scale-based GRIm-Score and survival outcomes remained significant. Both the total cohort and PSM cohort were subjected to multivariable analysis, which demonstrated that the three-category risk assessment-based GRIm-Score was a valuable predictor of both OS and PFS.

**Conclusions:**

In addition, the GRIm-Score may serve as a valuable and non-invasive prognostic predictor for SCLC patients undergoing PD1/PD-L1 immunotherapy.

## 1 Background

Approximately 15% of lung tumors are classified as the aggressive tumor type, small cell lung cancer (SCLC), which is traditionally classified into two stages: limited stage (LS-SCLC) and extensive stage (ES-SCLC) (1, 2). Meanwhile, immunotherapy has made significant advancements in the treatment of SCLC to this point (3–6). As the treatment for extended-stage SCLC in 2022, PD-1 inhibitors in combination with chemotherapy demonstrated a useful survival benefit, according to the most recent findings of the ASTRUM-005 research (7). At the same time, atezolizumab (PD-L1 inhibitor) plus carboplatin and etoposide (CP/ET) followed by maintenance atezolizumab was found to improve both overall survival (OS) and progression-free survival (PFS) compared to placebo plus CP/ET followed by maintenance placebo in the IMpower133 study (8). Although the introduction of the above-mentioned immunotherapies has improved the treatment of lung cancer, only a limited percentage of SCLC patients can benefit from this strategy. Therefore, there is still room for improvement in the long-term prognosis of SCLC patients undergoing immune checkpoint inhibitor (ICI) immunotherapies. However, a deeper understanding of prognostic indicators would help clinicians precisely identify potential patients who have a higher likelihood of having negative outcomes and establish a treatment plan.

Traditional cancer prognostic prediction generally focuses on pathological characteristics, like the stage of tumor node metastasis (TNM) (9). However, people with the common stage and identical treatment options have very different survival rates (10–12). In addition, a number of clinical risk scoring systems, including the prognostic nutritional index (PNI), the lung immune prognostic index (LIPI) score, and the systemic immune-inflammation index (SII), have also provided objective data for the prognostic prediction of immunotherapy lung cancer patients. These results prompt the development of a cutting-edge risk score system that offers clinicians prognostic prediction and risk classification (13–15). Since immune checkpoint inhibitors are being introduced to phase I trials, it has been reported that Gustave Roussy Immune Score (GRIm-Score) can be used as a quick and risk scoring system for predicting treatment prognoses of advanced-stage cancer and metastatic disease in clinical courses (14–16). The GRIm-Score, which incorporates the neutrophil-to-lymphocyte ratio (NLR), lactate dehydrogenase (LDH) level, and albumin level, has been shown to be useful for predicting survival in many types of cancer, such as pulmonary adenocarcinoma, breast cancer, and melanoma (13, 14, 17). Nevertheless, the predictive usefulness of GRIm-Score for SCLC patients undergoing immunotherapy treatment in actual clinical settings is still unknown. Therefore, the purpose of this retrospective article was to mainly determine the GRIm-Score values in SCLC patients receiving ICIs and determine whether it is a suitable index as a potential predictive biomarker.

## 2 Methods

### 2.1 Study participants

**Figure 1:**
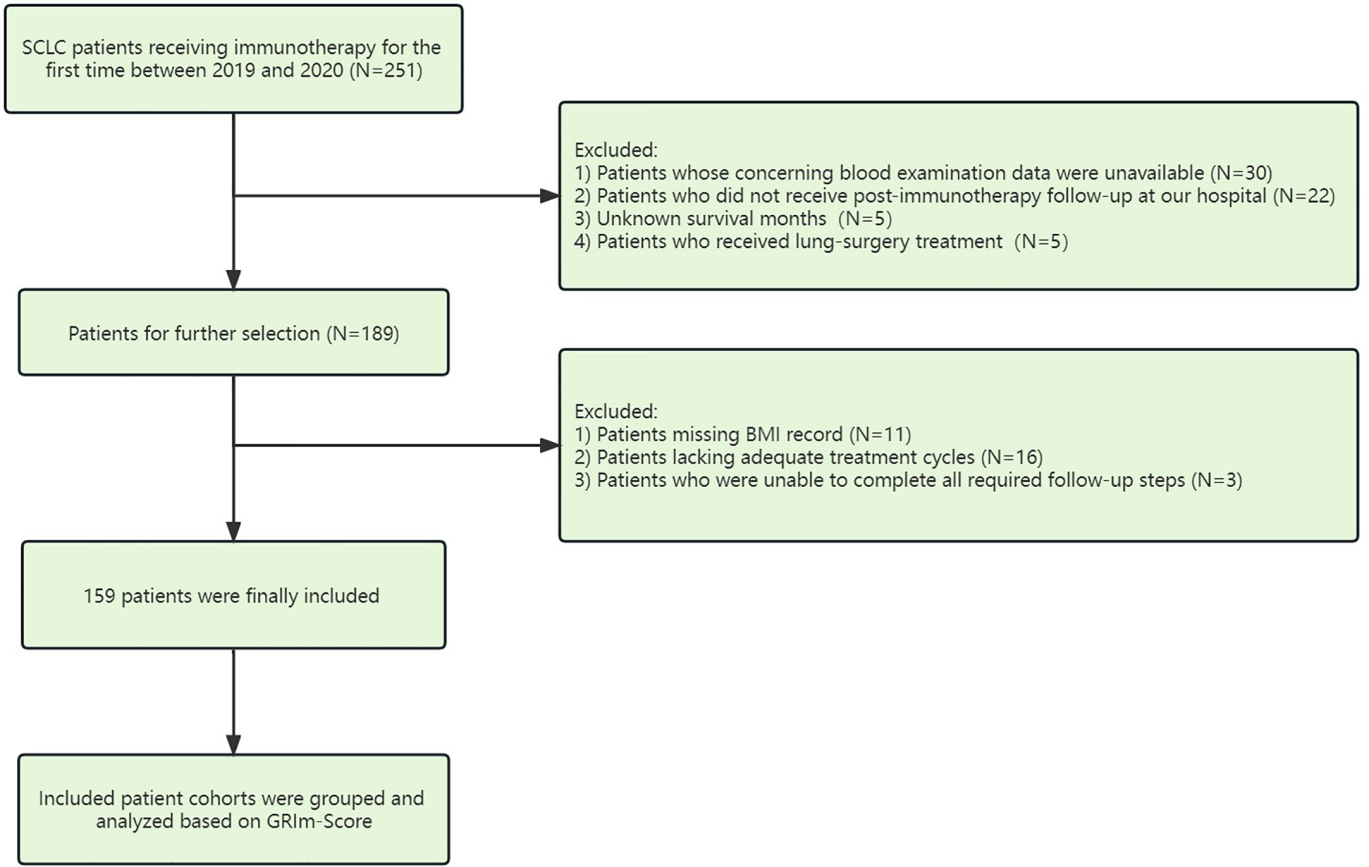
Flow diagram for the exclusion procedure of patients.

We reviewed the survival data of SCLC patients treated with immune checkpoint inhibitors from Shandong Cancer Hospital and Institute between January 2019 and December 2020. The following were the inclusion requirements for participants: 1) based on imaging characteristics, serological molecular markers, and high-risk factors, the clinical diagnosis of small cell lung cancer was made in accordance with the recommended guidelines. 2) Patients received ICI treatment for at least two courses. 3) Enrolled patients were aged 18 years and older. 4) Patients had good performance status and cardiovascular and pulmonary organ function. Moreover, **Figure 1** displays a thorough flow diagram of the patient exclusion procedure. We collected the specific clinicopathologic features and clinical reactions of the recruited individuals by reviewing their electronic medical records in retrospect. The Shandong Cancer Hospital and Institute’s institutional review board gave its approval for this study, which was carried out in conformity with the Declaration of Helsinki.

### 2.2 Data collection

**Figure 2:**
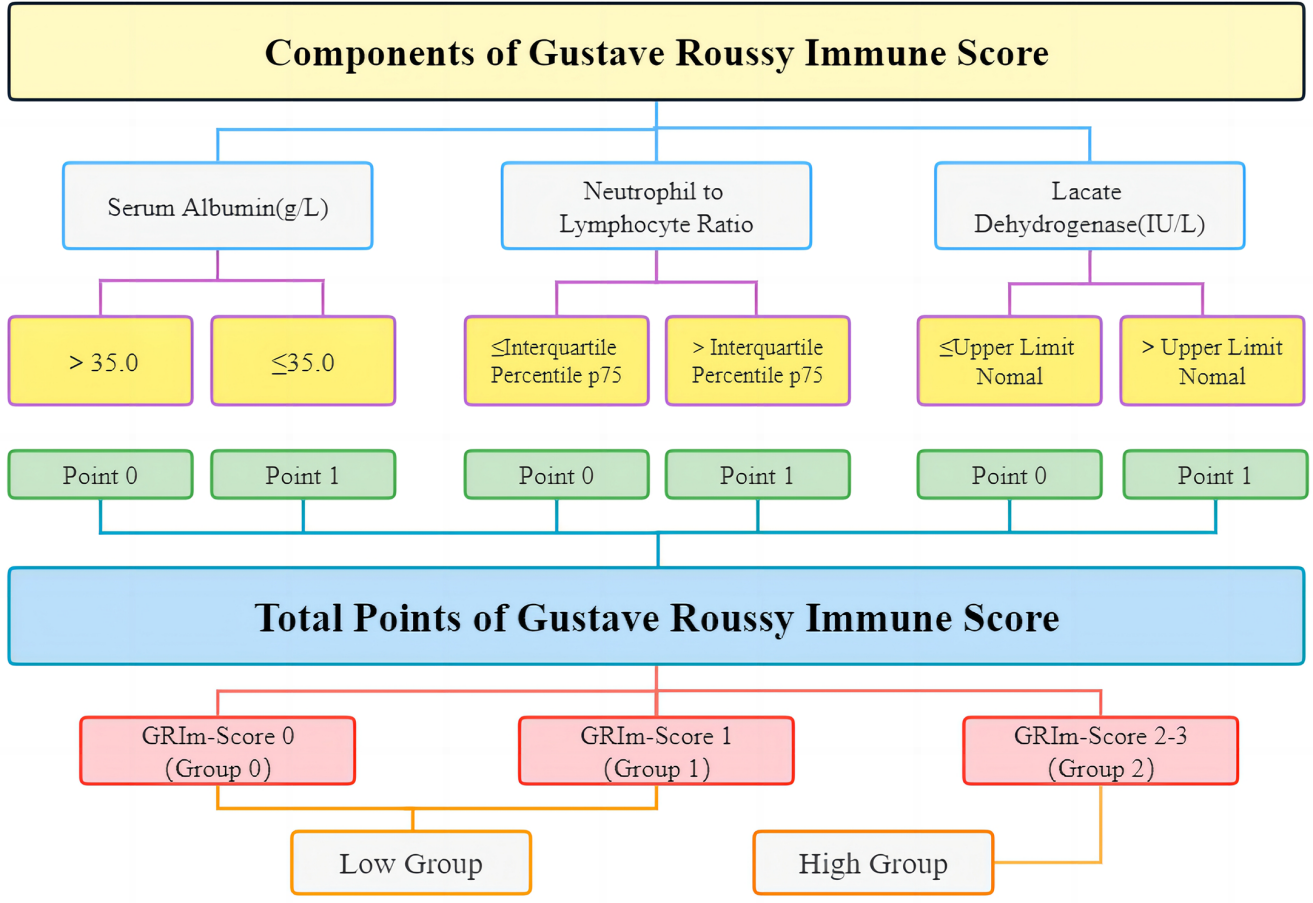
The detailed definition and grouping items of GRIm-Score. GRIm-Score, Gustave Roussy Immune Score.

The data included the project name, time of approval, the amount of funding, project type, application institution, and subject code. Retrospective data collection from our medical records included demographic indicators (age, sex, history of drinking, history of smoking, body mass index (BMI), and performance status), tumor characteristics (stage, metastasis, chemotherapy, and radiotherapy), and laboratory markers. Albumin, NLR, and LDH were combined to generate the most significant index (GRIm-Score). **Figure 2** offers a lucid depiction of the GRIm-Score tool’s definition and classification criteria. The GRIm-Score is calculated based on three parameters, namely, albumin (ALB), LDH, and NLR. Each variable is assigned a score of either 0 or 1 based on specific cutoff values. For instance, ALB levels of ≥35 g/L are scored as 0, while those <35 g/L are scored as 1. Similarly, normal LDH levels are scored as 0, whereas those above the upper limit of normal (ULN) of each center (245 U/L in this hospital) are scored as 1. For NLR, values ≤interquartile percentile p75 are scored as 0, and those >interquartile percentile p75 (2.7 in this hospital) are scored as 1. The sum of the scores for each variable yields a total GRIm-Score of 0, 1, 2, or 3, which is used to classify patients into three distinct categories: Group 0 (GRIm-Score 0), Group 1 (GRIm-Score 1), and Group 2 (GRIm-Score 2 or 3). Additionally, the study further stratified patients into two cohorts, a high-risk group (Group 2) and a low-risk group (Group 0 and Group 1), to facilitate a more refined analysis.

### 2.3 Response evaluation

Patients underwent follow-up imaging and serological tests every 6 weeks during treatment. Response Evaluation Criteria in Solid Tumors (RECIST) 1.1 was used to evaluate therapeutic outcomes. Complete response (CR), partial response (PR), stable disease (SD), and progressive disease (PD) were the four types of objective tumor responses. The primary outcome was OS and PFS. The time period from the initial administration of immune medicines until verified disease progression or mortality from any cause was referred to as PFS. Then, the length of OS was measured from the start of ICIs to the date of cancer-related death or loss to follow-up. The last follow-up date was 31 December 2020.

### 2.4 Statistical analysis

According to the kind of data, population demographics, clinical characteristics, and tumor characteristics from the categorical data examined were described as patient number with a percentage. The Kaplan–Meier (KM) analysis was utilized for survival analyses, and the log-rank test was used to compare survival rates. In order to examine variables that might be connected to treatment response and prognosis, uni- and multivariate Cox regression models were used. Then, a multivariate analysis was performed on variables with a p-value of less than 0.1 in univariate analyses. In the propensity score matching (PSM) analysis, which was applied to reduce the probable selection bias, caliper matching was used to match nearest neighbors, where the distance was set at 0.20 SD of the logit of propensity scores. Statistical analyses were completed with SPSS version 24.0 software, R version 4.2.4 statistical software, and STATA. Statistical analysis was conducted at a 0.05 significance level.

## 3 Results

### 3.1 Basic clinical information

Finally, 159 cases of SCLC patients who underwent immunotherapy were contained in our analysis based on pertinent inclusion criteria between January 2019 and December 2020 in **Figure 1**. With a mean age of 59.2 ± 9.7 years and a mean BMI of 25.1 ± 3.6 kg/m^2^, our cohort consists of 36 female and 123 male patients (male:female ratio = 77.4%:22.6%). Of the patients, 83 had a history of current or previous smoking (ratio = 52.2%), and the number was 100 (62.9%) when it comes to drinking. Of the patients, 68 (ratio = 42.8%) had lung radiotherapy (RT), and 131 of the patients (ratio = 82.4%) had chemotherapy prior to ICI therapy. Limited-stage and extensive-stage diagnoses were made in 100 (62.9%) and 59 (37.1%) individuals, respectively. According to pathologic criteria, extrathoracic metastasis was proven in 122 patients (ratio = 76.7%). Group 0 has 81 patients with a GRIm-score of 0. Group 1 and Group 2 have 58 and 20 patients, respectively, with GRIm-scores of 1 and 2, respectively. In contrast, 20 patients (12.5%) were assigned to the high-score group, and 139 patients (87.5%) were assigned to the low-score group (**Table 1**).

**Table 1:** Patient characteristics of the entire study cohort in three GRIm-Score groups.

Characteristics	Group 0	Group 1	Group 2	Total	p-Value
Age (years)					0.178
≤55	46 (56.8%)	37 (63.8%)	8 (40%)	91 (57.2%)	
>55	35 (43.2%)	21 (36.2%)	12 (60%)	68 (42.8%)	
Gender					0.68
Male	62 (76.5%)	44 (75.9%)	17 (85%)	123 (77.4%)	
Female	19 (23.5%)	14 (24.1%)	3 (15%)	36 (22.6%)	
Smoke					0.677
No	41 (50.6%)	27 (46.6%)	8 (40%)	76 (47.8%)	
Yes	40 (49.4%)	31 (53.4%)	12 (60%)	83 (52.2%)	
Drink					0.029
No	59 (72.8%)	31 (53.4%)	10 (50%)	100 (62.9%)	
Yes	22 (27.2%)	27 (46.6%)	10 (50%)	59 (37.1%)	
BMI					0.963
≤24	40 (49.4%)	30 (51.7%)	10 (50%)	80 (50.3%)	
>24	41 (50.6%)	28 (48.3%)	10 (50%)	79 (49.7%)	
Stage					0.411
Limited stage	47 (58%)	40 (69%)	13 (65%)	100 (62.9%)	
Extensive stage	34 (42%)	18 (31%)	7 (35%)	59 (37.1%)	
PS					0.363
≤80	45 (55.6%)	30 (51.7%)	14 (70%)	89 (56%)	
>80	36 (44.4%)	28 (48.3%)	6 (30%)	70 (44%)	
Lung RT before immunotherapy					0.189
No	40 (49.4%)	22 (37.9%)	6 (30%)	68 (42.8%)	
Yes	41 (50.6%)	36 (62.1%)	14 (70%)	91 (57.2%)	
EP chemotherapy before					0.23
No	12 (14.8%)	14 (24.1%)	2 (10%)	28 (17.6%)	
Yes	69 (85.2%)	44 (75.9%)	18 (90%)	131 (82.4%)	
Extrathoracic metastasis					0.07
No	13 (16%)	19 (32.8%)	5 (25%)	37 (23.3%)	
Yes	68 (84%)	39 (67.2%)	15 (75%)	122 (76.7%)	
Sintilimab					0.567
No	53 (65.4%)	42 (72.4%)	15 (75%)	110 (69.2%)	
Yes	28 (34.6%)	16 (27.6%)	5 (25%)	49 (30.8%)	
Immunotherapy type					0.208
PD-L1	29 (35.8%)	13 (22.4%)	5 (25%)	47 (29.6%)	
PD-1	52 (64.2%)	45 (77.6%)	15 (75%)	112 (70.4%)	
Erythrocyte					0.047
Normal ≥4.3	46 (56.8%)	32 (55.2%)	17 (85%)		
Abnormal **<**4.3	35 (43.2%)	26 (44.8%)	3 (15%)		
Hemoglobin					0.006
Normal ≥130	48 (59.3%)	32 (55.2%)	4 (20%)	84 (52.8%)	
Abnormal **<**130	33 (40.7%)	26 (44.8%)	16 (80%)	75 (47.2%)	
Platelet					0.172
Normal **>**125	75 (92.6%)	49 (84.5%)	16 (80%)	140 (88.1%)	
Abnormal **<**125	6 (7.4%)	9 (15.5%)	4 (20%)	19 (11.9%)	
Creatinine					0.038
Normal ≥45	74 (93.7%)	46 (80.7%)	19 (95%)	139 (89.1%)	
Abnormal **<**45	5 (6.3%)	11 (19.3%)	1 (5%)	17 (10.9%)	
Fe					0.034
Normal ≥9	74 (91.4%)	47 (81%)	14 (70%)	135 (84.9%)	
Abnormal **<**9	7 (8.6%)	19 (19%)	6 (30%)	24 (15.1%)	
K					0.435
3.5 ≤ Normal ≤ 5.5	77 (95.1%)	52 (89.7%)	19 (95%)	148 (93.1%)	
Abnormal **<**3.5 or **>**5.5	4 (4.9%)	6 (10.3%)	1 (5%)	11 (6.9%)	
Fibrinogen					0.015
Normal **≤**4	63 (77.8%)	39 (67.2%)	9 (45%)	111 (69.8%)	
Abnormal **>**4	18 (22.2%)	19 (32.8%)	11 (55%)	48 (30.2%)	

GRIm-Score, Gustave Roussy Immune Score; BMI, body mass index; RT, radiotherapy; EP, etoposide plus either cisplatin or carboplatin; PS, performance status.

In the GRIm-Score group, each number increase displayed substantial associations with erythrocyte, hemoglobin, creatinine, Fe, and fibrinogen when it came to peripheral laboratory markers as well. The remaining clinicopathologic factors did not significantly differ across the GRIm-Score groups (**Table 1**).

**Figure 3:**
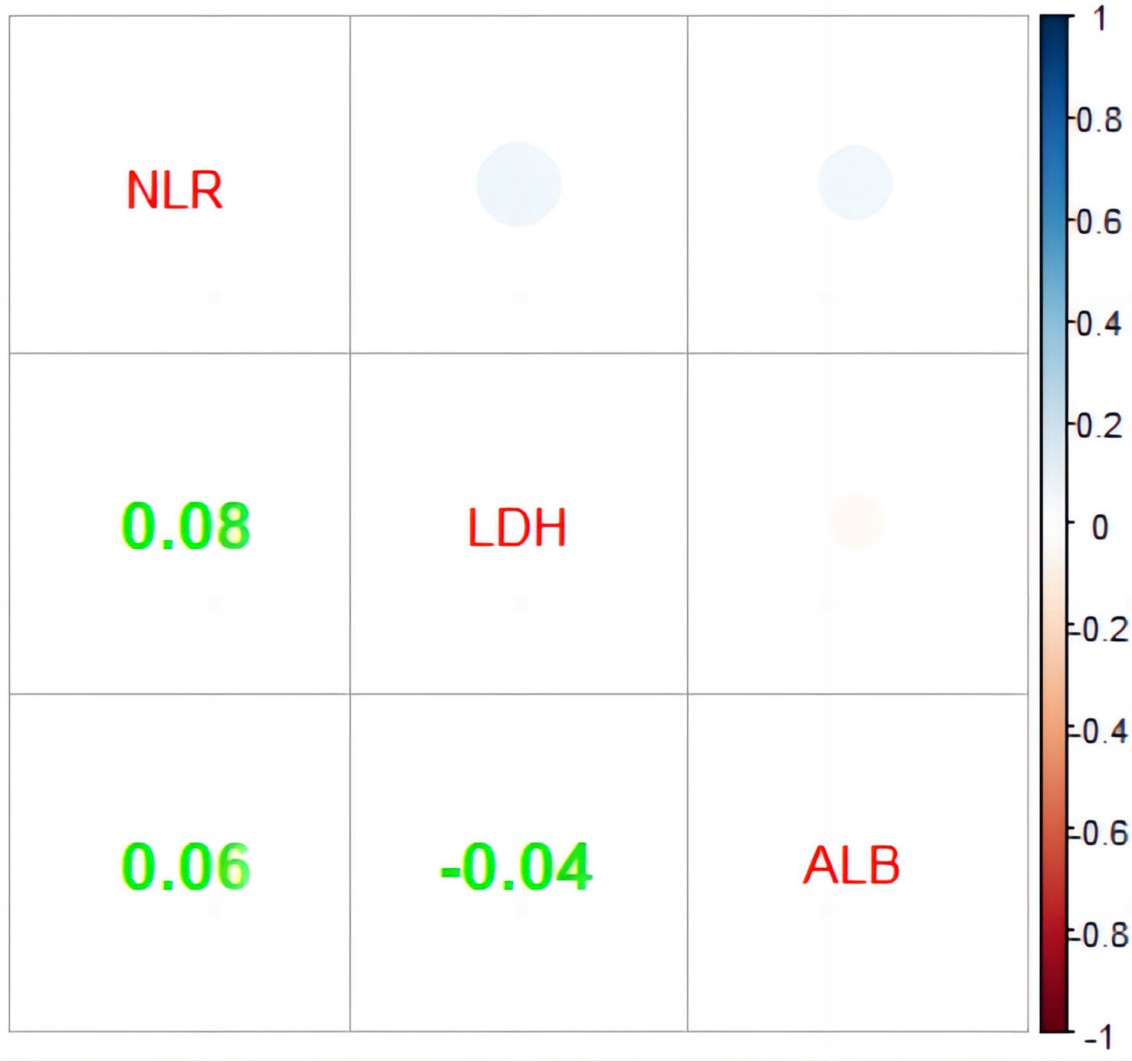
Correlation analysis of NLR, LDH, and ALB. NLR, neutrophil-to-lymphocyte ratio; LDH, lactate dehydrogenase (IU/L); ALB, albumin (g/L).

As part of the score, it is useful to assess the relation of NLR, LDH, and ALB, which is shown in **Figure 3**. In order to evaluate the link between these indicators, Spearman’s correlation analysis was used as shown. Unfortunately, no obvious correlations were found among them.

SD occurred in 84 patients, with a morbidity rate of 52.8% overall. There were no patients reaching CR when considering the time in all statistics. In addition, the rates of objective response and disease control were, respectively, 22.6% (n = 36) and 75.5% (n = 120) (**Table 2**).

**Table 2:** Response evaluation between the three GRIm-Score groups of the entire cohort.

Characteristics	Group 0	Group 1	Group 2	Total	p-Value
Objective response, n (%)					0.01
CR	0 (0%)	0 (0%)	0 (0%)	0 (0%)	
PR	21 (25.9%)	12 (20.7%)	3 (15%)	36 (22.6%)	
SD	39 (48.1%)	35 (60.3%)	10 (50%)	84 (52.8%)	
PD	20 (24.7%)	11 (19%)	4 (20%)	35 (22%)	
NE	1 (1.2%)	0 (0%)	3 (15%)	4 (2.5%)	
Objective response rate (%)	21 (25.9%)	12 (20.7%)	3 (15%)	36 (22.6%)	0.524
Disease control rate (%)	60 (74.1%)	47 (81%)	13 (65%)	120 (75.5%)	0.326

GRIm-Score, Gustave Roussy Immune Score; CR, complete response; PR, partial response; SD, stable disease; PD, progressive disease; NE, inevaluable.

### 3.2 Survival outcomes

**Figure 4:**
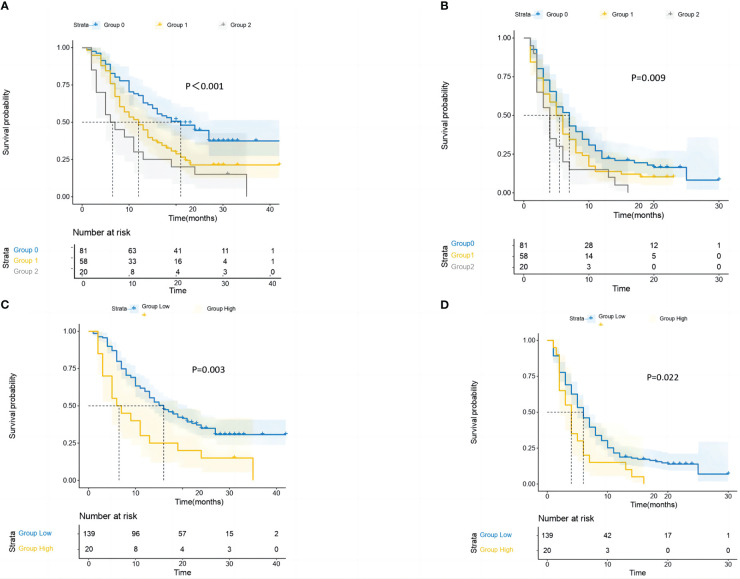
The study conducted survival probability analyses of OS and PFS based on the GRIm-Score estimated by a three-category risk assessment scale **(A**, **B)** and the original Bigot’s group **(C**, **D)**. The results are presented in the article. OS, overall survival; PFS, progression-free survival; GRIm-Score, Gustave Roussy Immune Score.

Strong relationships between the GRIm-Score Group 0–2 and post-immunotherapy survival up to the final follow-up duration were found by the Kaplan–Meier survival analysis (**Figure 4**). In the three groups, the mid-OS time was 21 (95% CI = 13.1–28.9), 12 (95% CI = 7.7–16.3), and 6 (95% CI = 1.6–10.3) months. Additionally, the median PFS for the three groups was 7 months (95% CI = 5.2–8.7), 5 months (95% CI = 3.8–6.1), and 4 months (95% CI = 2.9–5), respectively. Then, in the GRIm-Score group, the KM survival analyses showed that both PFS and OS were reduced with each increase in number (log-rank p < 0.001 and log-rank p = 0.009). Then, the KM survival analysis also demonstrated that the high-score cohort (median PFS, 4 months; median OS, 6 months) had worse PFS (log-rank p = 0.022) and OS (log-rank p = 0.003) when compared with the low-score cohort (median OS, 16 months; median PFS, 6 months).

To further identify the independent risk factors for ICI patients, we also used multivariate regression and univariate Cox regression (**Table 3**). Drinking history (p = 0.006), smoking history (p = 0.007), PS (p = 0.055), extrathoracic metastasis (p = 0.043), and fibrinogen (p = 0.009) were all significant factors in the univariable Cox regression analysis for the complete population GRIm-Score (three groups: Group 1 *vs.* Group 0, p = 0.01; Group 2 *vs.* Group 0, p < 0.001; former Bigot’s group: high group *vs.* low group, p = 0.005) were both substantially linked to worse post-therapy OS. Furthermore, the model of multivariable Cox regression developed on the three-category risk assessment-based GRIm-Score (model A) revealed that the new score (GRIm-Score Group 1 *vs.* GRIm-Score Group 0: HR = 1.846; 95% CI = 1.191–2.861; p = 0.006; GRIm-Score Group 2 *vs.* GRIm-Score Group 0: HR = 2.61; 95% CI = 1.485–4.589; p < 0.001), smoking history (HR = 1.814; 95% CI = 1.191–2.861; p = 0.029), extrathoracic metastasis (HR = 1.842; 95% CI = 1.106–3.047; p = 0.019), and fibrinogen (HR = 1.652; 95% CI = 1.006–2.487; p = 0.016) could all function as independent prognostic factors for poor OS of patients receiving ICI. However, in the multivariable Cox regression model, only GRIm-Score (group low *vs.* high: HR = 2.002; 95% CI = 1.187–3.861; p = 0.009) and fibrinogen (HR = 1.696; 95% CI = 1.136–2.544; p = 0.011) were statistically significant prognostic biomarkers for post-ICI OS by using the original Bigot’s group (model B). When examined separately in model A or B in multivariable Cox regression, the other peripheral hematologic indicators did not significantly affect prognosis.

**Table 3:** Prognostic factors for OS and PFS of SCLC patients in the entire cohort.

Characteristics	Univariable analysis (OS)		Multivariable analysis (A)		Multivariable analysis (B)		Univariable analysis (PFS)		Multivariable analysis (C)		Multivariable analysis (D)	
	HR (95% CI)	p-Value	HR (95% CI)	p-Value	HR (95% CI)	p-Value	HR (95% CI)	p-Value	HR (95% CI)	p-Value	HR (95% CI)	p-Value
Age (years)												
≤55												
>55	1.216 (0.792–1.865)	0.372					1.357 (0.863–1.784)	0.244				
Gender												
Male												
Female	0.682 (0.419–1.109)	0.123					0.783 (0.517–1.187)	0.249				
Smoke												
No												
Yes	1.707 (1.161–2.510)	0.007	1.814 (1.062–3.101)	0.029	1.649 (0.986–2.76)	0.057	1.122 (0.804–1.566)	0.499				
Drink												
No												
Yes	1.719 (1.17–2.524)	0.006	1.184 (0.695–2.016)	0.535	1.379 (0.832–2.285)	0.212	1.252 (0.888–1.767)	0.2				
BMI												
≤24												
>24	0.774 (0.53–1.129)	0.183					0.701 (0.5–0.982)	0.039	0.742 (0.521–1.057)	0.099	0.737 (0.517–1.05)	0.091
Stage												
Limited stage												
Extensive stage	1.144 (0.779–1.68)	0.492					1.414 (1.006–1.988)	0.046	0.993 (0.685–1.441)	0.972	0.964 (0.664–1.4)	0.847
PS												
≤80												
>80	0.684 (0.465–1.008)	0.055	0.706 (0.476–1.047)	0.084	0.707 (0.477–1.05)	0.086	0.605 (0.431–0.851)	0.004	0.682 (0.476–0.975)	0.036	0.679 (0.475–0.97)	0.033
Lung RT before immunotherapy												
No												
Yes	1.298 (0.883–1.908)	0.184					1.233 (0.88–1.727)	0.223				
EP chemotherapy before												
No												
Yes	1.264 (0.848–1.886)	0.25					1.066 (0.686–1.657)	0.777				
Extrathoracic metastasis												
No												
Yes	1.669 (1.017–2.739)	0.043	1.842 (1.106–3.067)	0.019	1.577 (0.957–2.599)	0.074	1.871 (1.218–2.875)	0.004	2.123 (1.339–3.365)	0.001	1.991 (1.264–3.137)	0.003
Sintilimab												
No												
Yes	0.996 (0.658–1.507)	0.983					0.855 (0.592–1.235)	0.404				
Immunotherapy type												
PD-L1												
PD-1	1.255 (0.827–1.905)	0.286					1 (0.697–1.437)	0.998				
Erythrocyte												
Normal ≥4.3												
Abnormal **<**4.3	0.855 (0.581–1.257)	0.426					0.851 (0.605–1.197)	0.353				
Hemoglobin												
Normal ≥130												
Abnormal **<**130	1.161 (0.798–1.69)	0.436					1.229 (0.88–1.716)	0.226				
Platelet												
Normal **>**125												
Abnormal **<**125	1.103 (0.605–2.011)	0.749					1.025 (0.608–1.726)	0.927				
Creatinine												
Normal ≥45												
Abnormal **<**45	1.08 (0.592–1.97)	0.801					0.935 (0.55–1.575)	0.8				
Fe												
Normal ≥9												
Abnormal **<**9	1.425 (0.848–2.394)	0.181					1.287 (0.807–2.055)	0.29				
K												
3.5 ≤ Normal ≤ 5.5												
Abnormal **<**3.5 or **>**5.5	1.113 (0.517–2.395)	1.113					0.976 (0.513–1.859)	0.942				
Fibrinogen												
Normal **≤**4												
Abnormal **>**4	1.692 (1.412–2.506)	0.009	1.652 (1.098–2.486)	0.016	1.696 (1.131–2.544)	0.011	1.51 (1.054–2.164)	0.025	1.367 (0.941–1.985)	0.101	1.414 (0.973–2.053)	0.069
GRIm-Score												
Group 0												
Group 1	1.717 (1.135–2.598)	0.01	1.846 (1.191–2.861)	0.006			1.319 (0.917–1.896)	0.136	1.441 (0.993–2.092)	0.05		
Group 2	2.61 (1.511–4.508)	<0.001	2.61 (1.485–4.589)	<0.001			1.93 (1.165–3.196)	0.011	1.832 (1.088–3.083)	0.023		
Original Bigot’s GRIm-Score group												
Low (score 0–1)												
High (score 2–3)	2.08 (1.251–3.457)	0.005			2.002 (1.187–3.376)	0.009	1.722 (1.068–2.777)	0.026			1.56 (0.955–2.547)	0.075

A and C: These multivariable Cox proportional hazards regression models were established on the three-category GRIm-Score risk assessment scale (0 vs. 1 vs. 2–3) with other clinicopathologic parameters with p < 0.10. B and D: These multivariable Cox proportional hazards regression models were established on the original Bigot**’**s GRIm-Score group (low vs. high) with other clinicopathologic parameters with p < 0.10.

CI, confidence interval; GRIm-Score, Gustave Roussy Immune Score; HR, hazard ratio; OS, overall survival; PFS, progression-free survival; SCLC, small cell lung cancer; BMI, body mass index; RT, radiotherapy.

In the same way, the three-category risk assessment-based GRIm-Score (model C) revealed that the GRIm-Score (Group 1 *vs.* Group 0: HR = 1.441; 95% CI = 0.993–2.092; p = 0.05; Group 2 *vs.* Group 0: HR = 1.832; 95% CI = 1.088–3.089; p = 0.023), PS (HR = 0.682; 95% CI = 0.476–0.975; p = 0.036), and extrathoracic metastasis (HR = 2.123; 95% CI = 1.339–3.367; p = 0.001) can function as independent prognostic factors for poor PFS of patients, while only PS (HR = 0.679; 95% CI = 0.475–0.97; p = 0.033) and extrathoracic metastasis (HR = 1.991; 95% CI = 1.264–3.137; p = 0.003) had the same trend by using the former Bigot’s group (model D). Additionally, we discovered no predictive relevance of a higher GRIm-Score as a result of post-ICI PFS when evaluating the GRIm-Score in accordance with the original Bigot’s group (**Table 3**).

In the receiver operating characteristic (ROC) analysis for the complete cohort, the three-category risk assessment scale of the GRIm-Score had an area under the ROC curve (AUC) of 0.639 (p = 0.005) and 0.622 (p = 0.085) for predicting OS and PFS, respectively. GRIm-Score’s AUC for predicting OS and PFS was 0.563 (p = 0.206) and 0.571 (p = 0.313), respectively, when it was estimated using the original Bigot’s group (**Supplementary Figures 1A, B**).

### 3.3 PSM cohort

When contrasting the reference concerning GRIm-Score Group 0 with the other groups, we found that there were appreciable differences in the drinking history and extrathoracic metastasis in **Table 1** (p < 0.1). In order to balance the confounding bias between the GRIm-Score Group 0 and the other groups, we used PSM analysis (**Supplementary Table 1**). As the result, our PSM procedure produced 51 and 20 well-matched couples between Groups 1 and 0, as well as between Groups 2 and 0. These PSM-derived cohorts had 20 overlapping matched pairs of patients, and a final cohort after PSM with 20 individuals in each GRIm-Score category with sufficiently comparable baseline features was created for additional studies, as shown in **Supplementary Table 1**.

Among the PSM cohort, we found that there existed a difference in objective response rate (45% *vs.* 10% *vs.* 15%; p = 0.018), especially when compared with the former no-PSM cohort (**Supplementary Table 2**).

### 3.4 Survival outcomes after PSM

**Figure 5:**
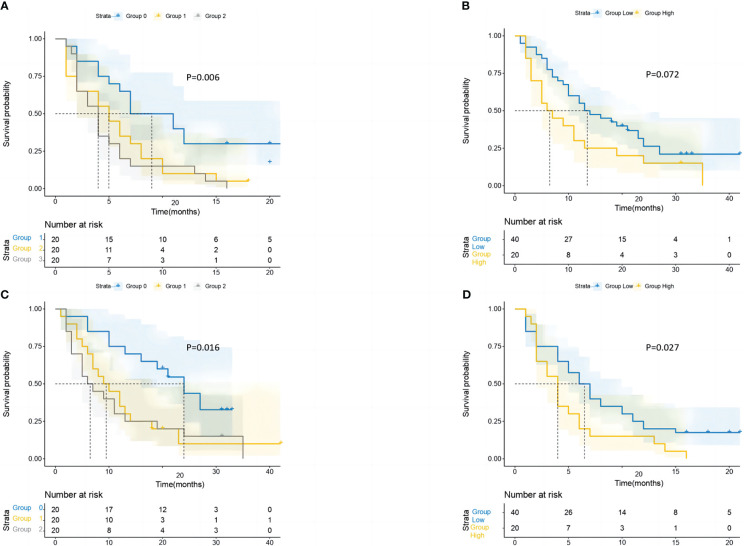
After performing PSM, the study conducted survival probability analyses of OS and PFS based on the GRIm-Score estimated by a three-category risk assessment scale **(A**, **C)** and the original Bigot’s group **(B**, **D)**. The results of these analyses are presented in the article. PSM, propensity score matching; OS, overall survival; PFS, progression-free survival; GRIm-Score, Gustave Roussy Immune Score.

GRIm-Score Groups 0, 1, and 2 in the PSM cohort had median OS of 24 (95% CI = 16.1–31.9), 9 (95% CI = 4.6–13.3), and 6 (95% CI = 1.6–10.3) months, respectively (**Figure 5**). Additionally, the median PFS times for the Groups 0, 1, and 2 were 7 (95% CI: 2.6–11.3), 5 (95% CI: 2.8–7.1), and 4 (95% CI = 2.9–5) months, respectively. Last but not least, the KM survival analyses performed on the cohort revealed that with each increase in the group’s number, both OS and PFS were considerably shortened. In addition, the KM survival analysis of the cohort showed that patients in the high-score group (median PFS, 4 months) had significantly shorter PFS (log-rank p = 0.027) than those in the low-score group (median PFS, 6 months). However, no significant difference was observed in OS (log-rank p = 0.072) of patients between the high-score group and the low-score group.

Similarly, we also employed univariate Cox regression along with multivariate regression to calculate the independent risk factors of ICI cohorts (**Table 4**). As a result, the model of multivariable Cox regression (model A) revealed that the GRIm-Score and smoking history could all function as independent prognostic factors for poor OS of patients. However, the multivariable Cox regression model used to estimate the GRIm-Score revealed only GRIm-Score to be a significant biomarker for post-ICI OS by using the former Bigot’s group (model B). When models A and B of multivariable Cox regression were examined separately, the other peripheral hematologic indicators did not significantly affect prognosis.

**Table 4:** Prognostic factors for overall survival and progression-free survival of the PSM cohort of SCLC patients.

Characteristics	Univariable analysis (OS)		Multivariable analysis (A)		Multivariable analysis (B)		Univariable analysis (PFS)		Multivariable analysis (C)		Multivariable analysis (D)	
	HR (95% CI)	p-Value	HR (95% CI)	p-Value	HR (95% CI)	p-Value	HR (95% CI)	p-Value	HR (95% CI)	p-Value	HR (95% CI)	p-Value
Age (years)												
≤55												
>55	1.047 (0.564–1.946)	0.884					1.252 (0.696–2.253)	0.453				
Gender												
Male												
Female	0.805 (0.398–1.631)	0.547					0.691 (0.354–1.349)	0.279				
Smoke												
No												
Yes	2.164 (1.163–4.028)	0.015	2.059 (1.021–4.152)	0.044	1.904 (0.947–3.824)	0.071	1.459 (0.843–2.524)	0.177				
Drink												
No												
Yes	1.499 (0.828–2.715)	0.181					1.287 (0.747–2.217)	0.364				
BMI												
≤24												
>24	0.604 (0.334–1.092)	0.095	0.654 (0.35–1.222)	0.183	0.593 (0.317–1.11)	0.103	0.652 (0.376–1.129)	0.127				
Stage												
Limited stage												
Extensive stage	1.639 (0.907–2.962)	0.102					1.325 (0.766–2.292)	0.314				
PS												
≤80												
>80	0.713 (0.39–1.304)	0.272					0,753 (0.433–1.308)	0.314				
Lung RT before immunotherapy												
No												
Yes	1.396 (0.782–2.492)	0.259					1.528 (0.889–2.627)	0.125				
EP chemotherapy before												
No												
Yes	1.07 (0.498–2.3)	0.863					0.976 (0.49–1.945)	0.945				
Extrathoracic metastasis												
No												
Yes	1.983 (0.921–4.269)	0.08	1.583 (0.694–3.697)	0.275	1.57 (0.687–3.584)	0.285	1.82 (0.933–3.55)	0.079	1.913 (0.976–3.751)	0.059	1.947 (0.99–3.831)	0.054
Sintilimab												
No												
Yes	1.07 (0.498–2.3)	0.863					0.701 (0.38–1.294)	0.256				
Immunotherapy type												
PD-L1												
PD-1	1.592 (0.82–3.091)	0.169					0.868 (0.481–1.567)	0.639				
Erythrocyte												
Normal ≥4.3												
Abnormal **<**4.3	0.786 (0.434–1.424)	0.428					0.742 (0.429–1.285)	0.287				
Hemoglobin												
Normal ≥130												
Abnormal **<**130	1.263 (0.707–2.255)	0.43					1.277 (0.744–2.194)	0.375				
Platelet												
Normal **>**125												
Abnormal **<**125	1.171 (0.495–2.767)	0.72					1.403 (0.628–3.135)	0.409				
Creatinine												
Normal ≥45												
Abnormal **<**45	0.904 (0.4–2.043)	0.809					0.58 (0.26–1.292)	0.182				
Fe												
Normal ≥9												
Abnormal **<**9	1.81 (0.892–3.673)	0.101					1.997 (1.009–3.925)	0.047	1.984 (0.967–4.078)	0.062	2.056 (1.005–4.293)	0.051
K												
3.5 ≤ Normal ≤ 5.5												
Abnormal **<**3.5 or **>**5.5	0.548	0.794 (0.374–1.686)					1.115 (0.518–2.4)	0.78				
Fibrinogen												
Normal **≤**4												
Abnormal **>**4	1.186 (0.637–2.211)	0.59					0.985 (0.548–1.773)	0.961				
GRIm-Score												
Group 0												
Group 1	2.473 (1.143–5.35)	0.021	2.38 (1.082–5.261)	0.031			2.272 (1.127–4.58)	0.022	2.25 (1.1–4.561)	0.025		
Group 2	2.683 (1.256–5.733)	0.011	3.2 (1.461–7.008)	0.004			2.698 (1.344–5.419)	0.005	2.504 (1.229–5.1)	0.011		
Original Bigot’s GRIm-Score group												
Low (score 0–1)												
High (score 2–3)	1.702 (0.935–3.097)	0.082			2.047 (1.104–3.789)	0.023	1.803 (1.025–3.173)	0.041			1.658 (0.928–2.96)	0.087

PSM, propensity score matching; SCLC, small cell lung cancer; OS, overall survival; PFS, progression-free survival; BMI, body mass index; RT, radiotherapy; GRIm-Score, Gustave Roussy Immune Score.

In the same way, the three-category risk assessment-based GRIm-Score (model C) revealed that the GRIm-Score can function as an independent prognostic factor for poor PFS of patients, while we discovered no predictive factors in terms of post-ICI PFS when evaluating the GRIm-Score in accordance with the original Bigot’s group (**Table 4**).

The three-category risk assessment scale of the GRIm-Score had an AUC of 0.717 (p = 0.014) and 0.823 (p = 0.006) for predicting OS and PFS, respectively, in the ROC analysis for the PSM cohort. GRIm-Score’s AUC for predicting OS and PFS was 0.624 (p = 0.162) and 0.689 (p = 0.107) when it was estimated using the original Bigot’s group (**Supplementary Figures 1C, D**).

## 4 Discussion

This study is the first to evaluate the predictive value of the GRIm-Score in SCLC patients undergoing PD1/PD-L1 immunotherapy treatment. The results suggest that the GRIm-Score can serve as a prognostic factor for both PFS and OS in this patient population. Based on the 159 patients mentioned above, we could draw the conclusion that an increase in GRIm-Score had a potential prognostic relevance for poor OS and PFS, which were comparable to those previously covered among patients with extensive stage non-small cell lung cancer (ES-NSCLC) receiving immunotherapy. Additionally, PSM analysis further confirmed that there were still such substantial correlations between ICI-SCLC GRIm-Score and survival outcomes in our study.

In fact, our research as a whole strongly demonstrated that the GRIm-Score might be used as a straightforward, non-invasive, and good discriminator for a clinical prognosis for small cell lung cancer patients receiving immunotherapy. An appropriate combination of the biological properties of the three peripheral hematologic indicators may help to clarify the potential causes of such obvious prognostic responsibilities concerning the immune score.

First of all, as a protein with a negative acute phase, in a clinical environment, the serum level of albumin is typically employed as a marker of patients’ nutritional status, as it reflects their nutritional state (18, 19). This indicates that both inflammation and hunger can lower the level of serum ALB (20). For instance, in patients with NSCLC receiving nivolumab therapy, the serum level of albumin may be a good clinical biomarker of 1-year survival and OS time (21). Additionally, a crucial prognostic and predictive sign for anti-PD-1 therapy in NSCLC patients was serum ALB level. These previous investigations suggested that a decreasing albumin level was a risk factor for both PFS and OS, which was consistent with the current findings.

Furthermore, an enzyme called lactate dehydrogenase, which is frequently present in many tissues throughout the human body and is also a well-known indicator of inflammation, plays a crucial part in anaerobic glycolysis and promotes cell proliferation (22). High LDH levels are a sign of poor overall survival in NSCLC because they are linked to the stimulation of tumor invasion and metastases (23–25). In addition, among patients treated with atezolizumab or docetaxel who had low or undetectable PD-L1 expression (TC0/IC0), an increased pretreatment LDH level was substantially linked to worse outcomes (26). As stated above, the application of LDH also has been confirmed in patients with advanced-stage NSCLC who received immunotherapy, although it is still unclear in patients with SCLC. However, LDH, as part of GRIm, was quite useful for judging the prognosis of immunotherapy SCLC patients in our study.

Third, cancer progression is characterized by inflammation, which is also a crucial element of the tumor microenvironment (27, 28). NLR has received widespread acceptance as an indicator of both tumor burden and systemic inflammatory response; the host immune system’s ability to inhibit carcinomatous angiogenesis can be severely compromised by a rapid decline in lymphocytes and excessive neutrophil activation, creating the ideal microenvironment for tumor progression (15, 19, 29–31). According to a recent study, NLR was an independent prognostic predictor in patients with advanced NSCLC who had nivolumab efficacy at baseline (8). NLR may therefore be able to predict survival in NSCLC patients undergoing immunotherapy, even though its role in ICI-treated SCLC patients is still unclear.

However, PSM analysis, which offers clear advantages over typical regression models to correct for observational research, was one of the study’s high points (32). Unbalanced variables in our study could result in selection bias because the three groups that were dichotomized above were not truly randomized. Therefore, we used a 1:1 PSM approach. In order to conduct further survival studies, a final PSM cohort was created, recruiting respectively 20 patients in three groups. At last, we found that the powerful predictive value of the GRIm-Score was successfully verified in the PSM cohort as well as remaining strongly dependable across the entire cohort. As a result, our conclusion drawn from the PSM analysis was more accurate and solid.

It should be noted that this study has a number of potential drawbacks that should not be disregarded.

First, the limitations of a single-center retrospective cohort study without external validation are inherent and should be considered when interpreting the results of the current investigation. Even though we made an effort to remove any potential confounding factors by using useful statistical methods of PSM and quite strict qualification requirements for the patients included, our results could still be affected by a number of selection biases, and less data could also weaken the demonstrative ability. Therefore, more sizable prospective validating investigations are needed in the future, with greater control over most of the evaluated clinicopathologic variables.

Then, a lengthy observation period might have altered the current results given that the study’s observation period was insufficient.

Last but not least, in this investigation, the GRIm-Score value was assessed throughout a single stage of immunotherapy. Studying the variations in this index during the immunotherapy follow-up period would also be substantially relevant. Future research paths were thought to focus on a prospective verification analysis of the dynamic prediction function of GRIm-Score in immunotherapy SCLC patients.

## 5 Conclusions

In general, the current study concludes by showing that GRIm-Score, a unique inflammatory and nutritional risk scoring system, is a strong predictive predictor in SCLC patients receiving immunotherapy. Our findings had significant clinical implications for the risk classification of immunotherapy-treated SCLC patients. Patients with high preoperative GRIm-Score levels typically have worse survival results, so these patients may need more follow-up visits as well as more adjuvant radiotherapy or chemotherapy.

## Data availability statement

The original contributions presented in the study are included in the article/**Supplementary Material**. Further inquiries can be directed to the corresponding authors.

## Author contributions

JS and XH contributed to both the original draft writing and the review and editing of the manuscript. JS and XH were responsible for the formal analysis. XL and ZZ contributed to the data curation and investigation. ZZ and XZ were responsible for the methodology and supervision. JY and DC were responsible for the resources, funding acquisition, and project administration. All authors contributed to the article and approved the submitted version.

## Supplementary material

Supplementary Figure 1 ROC curves revealing the discriminatory power of two evaluation indicators for predicting **(A)** OS and **(B)** PFS in the entire cohort; ROC curves revealing the discriminatory power of two evaluation indicators for predicting **(C)** OS and **(D)** PFS in the PSM cohort.
