# Supplementary figures and images for: Gustave Roussy immune score is a prognostic marker in patients with small cell lung cancer undergoing immunotherapy: a real-world retrospective study
Source: Front Oncol. 2023 May 2;13:1195499. doi: 10.3389/fonc.2023.1195499 (PMC10187137; doi:10.3389/fonc.2023.1195499)

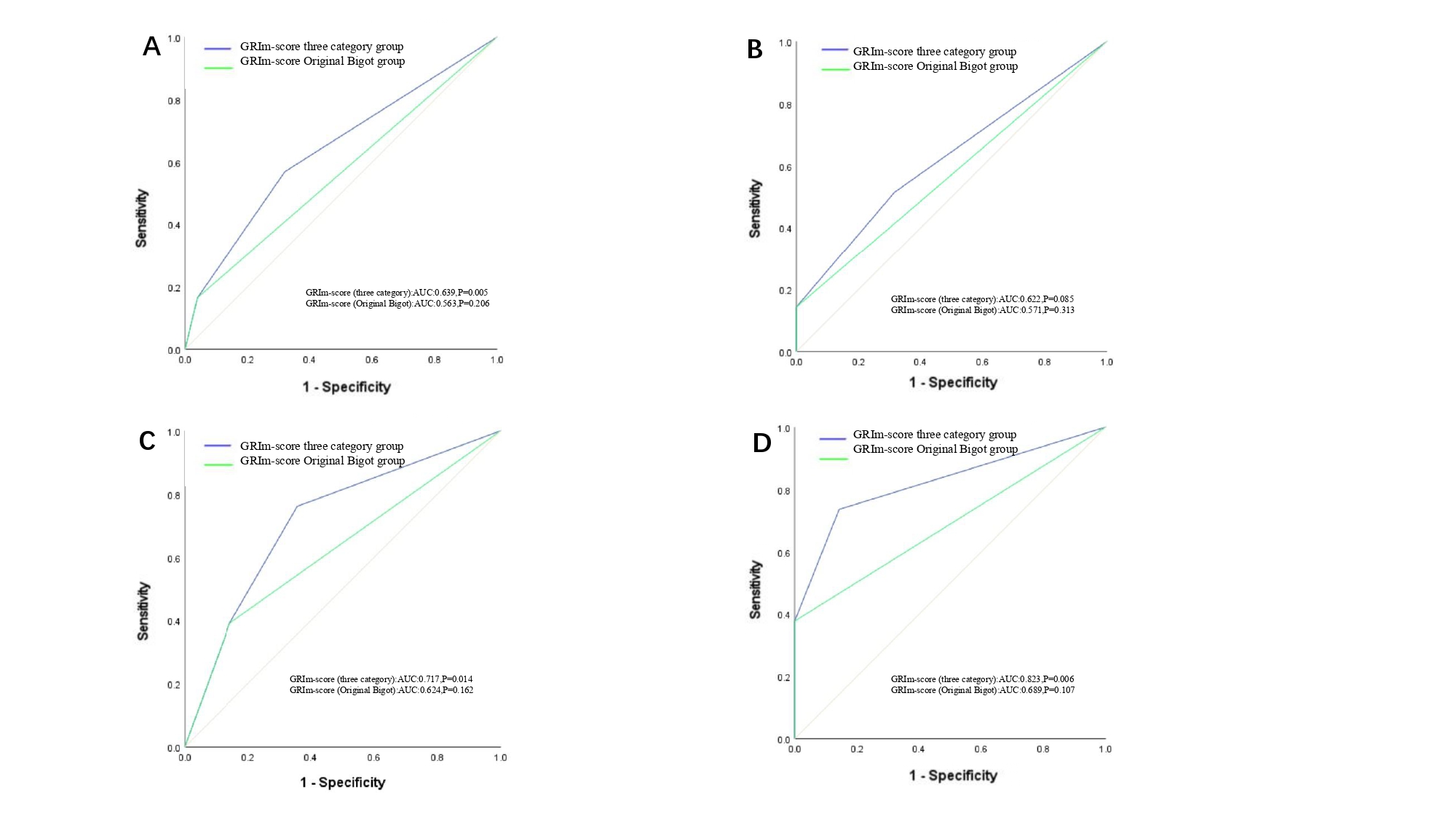

Supplement: Supplementary Figure 1 — ROC curves revealing the discriminatory power of two evaluation indicators for predicting (A) OS and (B) PFS in the entire cohort; ROC curves revealing the discriminatory power of two evaluation indicators for predicting (C) OS and (D) PFS in the PSM cohort. [file Image_1.jpeg]
