# Supplementary material for: Gustave Roussy immune score is a prognostic marker in patients with small cell lung cancer undergoing immunotherapy: a real-world retrospective study
Source: Front Oncol. 2023 May 2;13:1195499. doi: 10.3389/fonc.2023.1195499 (PMC10187137; doi:10.3389/fonc.2023.1195499)
Supplement: Supplementary file 2 [file Table_1.docx]

**Supplementary Table 1. Patient characteristics between the three GRIm-Score groups of the PSM cohort.**

| Characteristics | PSM-group 0 | PSM-group 1 | PSM-group 2 | Total | P |
| --- | --- | --- | --- | --- | --- |
| Age（y） |  |  |  |  | 0.183 |
| ≤55 | 13(65%) | 13(65%) | 8(40%) | 34(56.7%) |  |
| ＞55 | 7(35%) | 7(35%) | 12(60%) | 26(43.3%) |  |
| Gender |  |  |  |  | 0.449 |
| male | 14(70%) | 14(70%) | 17(85%) | 45(75%) |  |
| female | 6(30%) | 6(30%) | 3(15%) | 15(25%) |  |
| Smoke |  |  |  |  | 0.765 |
| No | 10(50%) | 10(50%) | 8(40%) | 28(46.7%) |  |
| Yes | 10(50%) | 10(50%) | 12(60%) | 32(53.3%) |  |
| Drink |  |  |  |  | 1 |
| No | 10(50%) | 10(50%) | 10(50%) | 30(50%) |  |
| Yes | 10(50%) | 10(50%) | 10(50%) | 30(50%) |  |
| BMI |  |  |  |  | 0.153 |
| ≤24 | 8(40%) | 14(70%) | 10(50%) | 32(53.3%) |  |
| ＞24 | 12(60%) | 6(30%) | 10(50%) | 28(46.7%) |  |
| Stage |  |  |  |  | 0.619 |
| limited stage | 10(50%) | 12(60%) | 13(65%) | 35(58.3%) |  |
| extensive stage | 10(50%) | 8(40%) | 7(35%) | 25(41.7%) |  |
| PS |  |  |  |  | 0.41 |
| ≤80 | 10(50%) | 11(55%) | 14(70%) | 35(58.3%) |  |
| ＞80 | 10(50%) | 9(45%) | 6(30%) | 25(41.7%) |  |
| Lung-RT before immunotherapy | | | |  | 0.005 |
| No | 16(80%) | 9(45%) | 6(30%) | 31(51.7%) |  |
| Yes | 4(20%) | 11(55%) | 14(70%) | 29(48.3%) |  |
| EP-chemotherapy before | | |  |  | 0.235 |
| No | 3(15%) | 6(30%) | 2(10%) | 11(18.3%) |  |
| Yes | 17(85%) | 14(70%) | 18(90%) | 49(81.7%) |  |
| Extrathoracic metastasis | | |  |  | 1 |
| No | 5(25%) | 5(25%) | 5(25%) | 15(25%) |  |
| Yes | 15(75%) | 15(75%) | 15(75%) | 45(75%) |  |
| sintilimab |  |  |  |  | 0.788 |
| No | 14(70%) | 13(65%) | 15(75%) | 42(70%) |  |
| Yes | 6(30%) | 7(35%) | 5(25%) | 18(30%) |  |
| immunotherapy type | |  |  |  | 0.72 |
| PD-L1 | 7(35%) | 5(25%) | 5(25%) | 17(28.3%) |  |
| PD-1 | 13(65%) | 15(75%) | 15(75%) | 43(71.7%) |  |
| Erythrocyte | |  |  |  | 0.012 |
| Nomal≥4.3 | 9(45%) | 9(45%) | 17(85%) | 35(58.3%) |  |
| Abnomal＜4.3 | 11(55%) | 11(55%) | 3(15%) | 25(41.7%) |  |
| Hemoglobin | |  |  |  | 0.001 |
| Nomal≥130 | 14(70%) | 14(70%) | 4(20%) | 32(53.3%) |  |
| Abnomal＜130 | 6(30%) | 6(30%) | 16(80%) | 28(46.7%) |  |
| Platelet |  |  |  |  | 0.562 |
| Nomal＞125 | 18(90%) | 18(90%) | 16(80%) | 52(86.7%) |  |
| Abnomal＜125 | 2(10%) | 2(10%) | 4(20%) | 8(13.3%) |  |
| Creatinine |  |  |  |  | 0.064 |
| Nomal≥45 | 18(90%) | 14(70%) | 19(95%) | 51(85%) |  |
| Abnomal＜45 | 2(10%) | 6(30%) | 1(5%) | 9(15%) |  |
| Fe |  |  |  |  | 0.112 |
| Nomal≥9 | 19(95%) | 15(75%) | 14(70%) | 48(80%) |  |
| Abnomal＜9 | 1(5%) | 5(25%) | 6(30%) | 12(20%) |  |
| K |  |  |  |  | 0.596 |
| 3.5≤Nomal≤5.5 | 20(100%) | 19(95%) | 19(95%) | 58(96.7%) |  |
| Abnomal＜3.5or＞5.5 | 0(0%) | 1(5%) | 1(5%) | 2(3.3%) |  |
| Fibrinogen | |  |  |  | 0.057 |
| Nomal≤4 | 16(80%) | 16(80%) | 10(50%) | 42(70%) |  |
| Abnomal＞4 | 4(20%) | 4(20%) | 10(50%) | 18(30%) |  |
| Original Bigot's GRIm-Score group | | | |  |  |
| Low (Score 0–1) | 20(100%) | 20(100%) | - | 40(66.7%) |  |
| High (Score 2–3) | - | - | 20(100%) | 20(33.3%) |  |
