# Supplementary material for: Gustave Roussy immune score is a prognostic marker in patients with small cell lung cancer undergoing immunotherapy: a real-world retrospective study
Source: Front Oncol. 2023 May 2;13:1195499. doi: 10.3389/fonc.2023.1195499 (PMC10187137; doi:10.3389/fonc.2023.1195499)
Supplement: Supplementary file 3 [file Table_2.docx]

**Supplementary Table2. Response evalution between the three GRIm-Score groups of the PSM cohort.**

| Characteristics | Group 0 | Group 1 | Group 2 | Total | P |
| --- | --- | --- | --- | --- | --- |
| Objective response, n (%) | | |  |  | 0.055 |
| CR | 0(%) | 0(0%) | 0(0%) | 0(0%) |  |
| PR | 9(45%) | 2(10%) | 3(15%) | 14(23.3%) |  |
| SD | 6(30%) | 13(65%) | 10(50%) | 29(48.3%) |  |
| PD | 4(20%) | 5(25%) | 4(20%) | 13(21.7%) |  |
| NE | 1(5%） | 0(0%) | 3(15%) | 4(6.7%) |  |
| Objective response rate (%) | 9(45%) | 2(10%) | 3(15%) | 14(23.3%) | 0.018 |
| Disease control rate (%) | 15(75%) | 15(75%) | 13(65%) | 43(71.7%) | 0.72 |
